# Validation of Self-Reported Sleep Against Actigraphy

**DOI:** 10.2188/jea.JE20120012

**Published:** 2012-09-05

**Authors:** Jennifer Girschik, Lin Fritschi, Jane Heyworth, Flavie Waters

**Affiliations:** 1 Western Australian Institute for Medical Research, The University of Western Australia, Nedlands, Australia; 2 School of Population Health, The University of Western Australia, Crawley, Australia; 3 Centre for Clinical Research in Neuropsychiatry, Graylands Hospital, and School of Psychiatry and Clinical Neurosciences, The University of Western Australia, Crawley, Australia

**Keywords:** sleep, sleep duration, sleep quality, validity, actigraphy

## Abstract

**Background:**

Self-report remains the most practical and cost-effective method for epidemiologic sleep studies involving large population-based samples. Several validated questionnaires have been developed to assess sleep, but these tools are lengthy to administer and may be impractical for epidemiologic studies. We examined whether a 3-item sleep questionnaire, similar to those typically used in epidemiologic studies, closely corresponded with objective measures of sleep as assessed using actigraphy monitoring.

**Methods:**

Eligible participants were Western Australian women aged 18 to 80 years. Participants completed a sleep questionnaire, wore a wrist actigraph for 7 nights, and completed a brief daily sleep log. Objective actigraphy measurements for 56 participants were summarized by mean and mode and compared with the subjective reports, using weighted kappa and delta.

**Results:**

Data collected from the questionnaire showed poor agreement with objectively measured sleep, with kappas ranging from −0.19 to 0.14.

**Conclusions:**

Our results indicate that sleep questions typically used in epidemiologic studies do not closely correspond with objective measures of sleep as assessed using actigraphy. The findings have implications for studies that have used such sleep questions. A means of appropriately measuring sleep as a risk factor in epidemiologic studies remains to be determined.

## INTRODUCTION

Evidence from laboratory studies has identified a number of plausible biological models by which sleep may influence long-term health outcomes.^1^ A number of epidemiologic studies have investigated self-reported sleep and long term health outcomes such as obesity, diabetes, and cancer,^2^^–^^13^ and most have reported a link between poor sleep and increased morbidity and mortality.^2^^,^^5^^–^^7^^,^^11^^–^^13^ However, the results of these studies may not be valid if self-reported sleep does not reflect actual sleep. Although polysomnography (PSG) is the gold standard for assessing sleep, self-report remains the most practical and cost-effective method for epidemiologic studies attempting to collect information on large population-based samples.

Several sleep questionnaires have been validated and show moderate correlations with objectively measured sleep parameters. These questionnaires include the Karolinska Sleep Diary, the Pittsburgh Sleep Diary, the Sleep Timing Questionnaire, the Athens Insomnia Scale, and the Pittsburgh Sleep Quality Index.^14^^–^^16^ However, such tools are lengthy to administer and may be inappropriate or impractical for epidemiologic studies. The compromise for many epidemiologic studies has been to limit assessment of self-reported sleep to 1 or 2 questions, most commonly those asking about usual sleep duration,^3^^,^^10^^,^^17^^,^^18^ and less commonly about usual subjective quality^12^^,^^13^ or ease of getting to sleep.^2^^,^^5^^,^^7^ However, these types of questions have not been validated against objective sleep measures, although 1 study validated its questionnaire against sleep diary information.^18^ In the present study, we examined whether the assessment of sleep using 3 questions typically used by epidemiologic studies corresponded to objective measures of sleep as assessed using actigraphy in a population of Western Australian women. Information derived from the questionnaire was assessed against data generated by the actigraph, which is a small biomedical instrument that provides objective multi-day recordings of sleep–wake periods. Actigraphy allows 24-hour recording in the home environment under “lived” conditions and has been recommended by the American Association of Sleep Medicine as an acceptably accurate estimate of sleep patterns.^19^ The questions used in this study were previously shown to be reliable.^20^

## METHODS

### Study population

A minimum sample size of 40 women was calculated using the Walter method.^21^ Participants were recruited through newspaper advertisements and from The University of Western Australia staff e-mail list calling for volunteers for a sleep study. Eligible participants were women aged 18 to 80 years who spoke adequate English to complete the questionnaire and had no self-reported history of a diagnosis of a sleep disorder (excluding transient insomnia). Men were excluded from this study because the questionnaire for validation was intended for use with female populations only, as it was developed for a study of breast cancer.

The study was approved by The University of Western Australia’s Human Research Ethics Committee and was performed in accordance with the Declaration of Helsinki.

### Data collection

Participants gave informed consent before completing a modified version of the Breast Cancer Environment and Employment Study (BCEES) sleep questionnaire, wearing an actigraph on their dominant wrist for 24 hours a day for 8 days (7 nights),^19^ and completing a brief daily sleep log.

The BCEES sleep questionnaire was developed for an ongoing case-control study of environmental and occupational risk factors for breast cancer. The questionnaire was designed to collect information on demographic characteristics (age, education, and employment), 2 domains of sleep (usual sleep duration and subjective sleep quality), and exposure to white light while sleeping. Information on duration was assessed separately for workdays and non-workdays, as people tend to catch-up on sleep on non-workdays. Specifically, the sleep questions were, “How many hours of sleep on work [non/work] days do you usually get?” and “Do you generally consider yourself to be a good sleeper, that is, do you fall asleep easily and sleep soundly?”.

Actigraphy uses wrist-worn accelerometers that measure gross motor activity, from which sleep/wake can be inferred. Actigraphy has been shown to correlate well with PSG in normal sleepers and is a more cost-effective and practical method of objective sleep monitoring for small population-based studies.^19^ Seven days of actigraphy recording has been shown to be sufficient to obtain stable measures of domains of sleep.^22^^,^^23^

The Actiwatch Spectrum (Philips Respironics, Murrysville, PA, USA) used in this study contains a light sensor that measures white light in lumens/m^2^ (lux) and an event marker button to indicate specific times. Consistent with standard procedure in actigraphy studies, participants were asked to press the event marker button when they turned out the lights to go to sleep at night, when they got out of bed in the morning, and if they woke during the night for any reason.

The output of the actigraph was digitally integrated using actigraphy principles. Sleep parameters were automatically scored using the manufacturer’s software (Actiware 5.59), with an epoch length of 30 seconds and a medium wake threshold value of 40 seconds. Measures were total sleep time in minutes, sleep onset latency in minutes (period between bed time and sleep onset), wake after sleep onset in minutes (time spent awake after initial onset of sleep), and efficiency (percentage of time in bed spent asleep).

Participants completed a daily sleep log to record details such as sleep and wake times, whether the day was a workday, whether they took any naps, and whether the watch was removed for any period. This information was used to cross-validate the actigraphy data. Coding of workdays and non-workdays was done manually on the basis of the sleep logs.

### Statistical analysis

To facilitate comparison between categorical questionnaire items and continuous actigraphy variables, actigraphy output was converted to categorical variables based on the mean and mode values.^24^ Below, we describe the calculation of the mean and mode values. Where there were 2 or more modes, the smallest mode was chosen for categorization.

#### Total sleep time

Mean: Actigraphic total sleep time was averaged separately for workdays and non-workdays and converted to 6-level categorical variables consistent with the answer categories for the sleep duration questionnaire item (<5 h, 5–6 h, 6–7 h, 7–8 h, 8–9 h, >9 h).

Mode: Actigraphic total sleep time for each night was converted to a 6-level categorical variable consistent with the answer categories for the sleep duration questionnaire item and the mode duration identified for workdays and non-workdays separately.

Because the first 2 and last 2 categories included very few participants, they were condensed into 4 categories for analysis (<6 h, 6–7 h, 7–8 h, and >8 h).

#### Sleep onset latency, sleep efficiency, wake after sleep onset, and quality of sleep

Mean: Sleep onset latency, efficiency, and wake after sleep onset were averaged for the nights of actigraphic data collection and converted to 4-level categorical variables consistent with the answer categories from the sleep quality questionnaire (ie, very good sleeper, fairly good sleeper, fairly bad sleeper, very bad sleeper) using the cut-points shown in Table 1, which were derived from the literature on normal and abnormal sleep habits.^25^^–^^29^ In addition, a composite objective sleep quality variable was created for comparison with the sleep quality question. The composite objective sleep quality score was created based on the mean sleep onset latency and either the mean efficiency or mean wake after sleep onset reaching the minimum (and not exceeding the maximum) cut-point for inclusion in that category (see Tables 1 and 2). For example, a participant with “fairly good” sleep onset latency and “very good” efficiency would be classified as having “very good” composite quality, while a person with “fairly good” sleep onset latency and “fairly good” efficiency would be classified as having “fairly good” composite quality.

**Table 1.** Cut-points for converting continuous variables collected by actigraphy to 4-level categorical variables consistent with the answer categories for the question on subjective sleep quality

Categorical variable	Sleep onset latency, min	Efficiency	Wake after sleep onset, min
Very good sleeper	<15	>90%	<5
Fairly good sleeper	15–20	85–90%	5–15
Fairly bad sleeper	21–31	80–84%	16–30
Very bad sleeper	>31	<80%	>31

**Table 2.** Criteria for creating a composite sleep quality variable based on participants meeting a quality threshold for sleep onset latency and either efficiency or wake after sleep onset as measured by actigraphy

Sleep onset latency	Efficiency or wake after sleep onset			

	Very good	Fairly good	Fairly bad	Very bad
Very good	Very good	Very good	Fairly good	Fairly bad
Fairly good	Very good	Fairly good	Fairly good	Fairly bad
Fairly bad	Fairly good	Fairly good	Fairly bad	Very bad
Very bad	Fairly bad	Fairly bad	Very bad	Very bad

Mode: Sleep onset latency, efficiency, and wake after sleep onset for each night were converted to 4-level categorical variables using the cut-points in Table 1 and the mode identified. A composite objective sleep quality score was created using a method similar to that described for the mean.

#### Sensitivity analysis for sleep onset latency, efficiency, wake after sleep onset, and quality of sleep

While the cut-points defined in Table 1 were defined using the literature, there is no formal standard definition of objective sleep quality. To investigate the sensitivity of the kappa statistic to changes in these cut-points, we created separate 4-level categorical variables for mean sleep onset latency, mean efficiency, and mean wake after sleep onset based on quartiles, and repeated the analysis. An additional composite objective sleep quality score was also created using a method similar to that described for the mean above.

Objective actigraphy measurements were compared to the questionnaire using kappa with quadratic weights in Stata 11 (StataCorp, College Station, TX, USA). The confidence intervals for kappa were obtained using the kapci command in Stata, utilizing bootstrap methods with 2000 replications. People without duration data were excluded from the duration analysis. Analysis of sleep duration was conducted with naps both included and excluded, but, because the results were not substantially different, only the results for sleep duration excluding naps are presented.

A limitation of the kappa statistic is that it is sensitive to the marginal distribution. If marginal totals are very small or very unbalanced, the resulting kappa can be paradoxically high or low as compared with the proportion of observed agreement.^30^^,^^31^ Delta is an alternative chance-corrected measure of validity that is not sensitive to marginal totals but will be similar to kappa when marginal totals are not excessively unbalanced.^31^ Because of the small numbers in this study, delta values were calculated in addition to kappa values using the program written by Martin and Femia.^31^

## RESULTS

Data collection occurred between 21 January and 30 March 2011. A total of 61 women participated and completed all parts of the study. However, a faulty watch compromised the data from 5 participants, which left 56 participants for analysis. All participants except 2 completed 7 nights of actigraphy. Eight participants were unemployed, 1 was on holiday, and 1 worked for the duration of the study, leaving 47 and 55 participants for the analysis of duration of sleep on workdays and non-workdays, respectively.

Age ranged from 22 to 78 years (mean, 46 years; Table 3). Most participants (57%) were born in Australia or New Zealand, 71% had completed high school, and 86% were employed either full- or part-time.

**Table 3.** Demographic characteristics of participants (*n* = 56)

Characteristics	*n*	%
Age (mean, range)	46 (22–78)	
Country/region of birth		
Australia/New Zealand	32	57
United Kingdom/Ireland	14	25
Continental Europe	2	4
Asia	1	2
Other	7	12
High school education		
≤Year 9 or equivalent	1	2
Year 10 or equivalent	7	13
Year 11 or equivalent	8	14
Year 12 or equivalent	40	71
Employment		
Full-time	28	50
Part-time	20	36
None	8	14

Table 4 shows the distributions for workday sleep duration and composite quality by questionnaire response and by the mean and mode of actigraphy data.

**Table 4.** Distribution of participants for workday duration and composite quality by questionnaire response and by mean and mode of actigraphy measurements

	Questionnaire items	Actigraphy items	
	
		Mean	Mode
			
	*n* (%)	*n* (%)	*n* (%)
Duration (workdays)			
6 or less hours	15 (32)	5 (11)	9 (19)
6–7 hours	16 (34)	13 (28)	10 (21)
7–8 hours	15 (32)	26 (55)	25 (53)
8 or more hours	1 (2)	3 (6)	3 (6)

Composite quality			
Very good	14 (25)	6 (11)	7 (13)
Fairly good	22 (39)	19 (34)	24 (43)
Fairly bad	16 (29)	19 (34)	19 (34)
Very bad	4 (7)	12 (21)	6 (10)

The BCEES sleep items showed poor agreement with objectively measured sleep habits as assessed using actigraphy (Table 5). In particular, kappa values were negative for the agreement between subjective and mean objective measures relating to duration and efficiency. The agreement between subjective quality and mean actigraphic sleep onset latency and wake after sleep onset was positive but very weak. A comparison of subjective sleep quality with the composite measure of objective quality showed slightly better agreement. The sensitivity analyses of the subjective and mean objective measures relating to sleep onset latency, efficiency, wake after sleep onset, and the composite measure of objective quality did not appreciably alter the results (data not shown).

**Table 5.** Weighted (quadratic) kappa scores and delta scores for agreement between subjective self-reports of sleep quality and objective measures of sleep quality as recorded by actigraphy

Usual sleep-habits question	Observed agreement (%)	Kappa		Delta	
	
		Kappa (95% CI)	*P*-value	Delta	*P*-value
**Mean**					
Duration of sleep on workdays (*n* = 47)	81%	−0.08 (−0.32 to 0.11)	0.76	−0.01	0.56
Duration of sleep on non-workdays (*n* = 55)	78%	−0.13 (−0.34 to 0.07)	0.86	0.00	0.14
Sleep onset latency (*n* = 56)	80%	0.08 (−0.16 to 0.33)	0.27	0.09	0.86
Sleep efficiency (*n* = 56)	79%	−0.19 (−0.39 to 0.01)	0.93	−0.24	0.64
Wake after sleep onset (*n* = 56)	76%	0.10 (−0.02 to 0.26)	0.08	0.05	0.61
Sleep quality (*n* = 56)	82%	0.14 (−0.08 to 0.36)	0.12	−0.11	0.92

**Mode**					
Duration of sleep on workdays (*n* = 47)	83%	0.05 (−0.19 to 0.29)	0.34	0.11	0.60
Duration of sleep on non-workdays (*n* = 55)	79%	0.01 (−0.28 to 0.27)	0.47	0.09	0.12
Sleep onset latency (*n* = 56)	76%	0.02 (−0.16 to 0.24)	0.40	0.05	0.77
Sleep efficiency (*n* = 56)	81%	−0.08 (−0.31 to 0.16)	0.74	0.04	0.67
Wake after sleep onset (*n* = 56)	75%	0.07 (−0.08 to 0.22)	0.17	0.09	0.82
Sleep quality (*n* = 56)	84%	0.09 (−0.17 to 0.33)	0.25	−0.15	0.89

The results did not substantially change when the mode of the sleep variables was used. However, the agreement for duration on workdays and non-workdays was slightly improved.

Participants with the shortest self-reported sleep durations tended to underestimate their sleep as compared with objective measures. In particular, all participants who self-reported a usual sleep duration of 6 hours or less recorded an objective mean sleep duration of greater than 6 hours. In contrast, participants who self-reported sleeping 8 hours or more tended to overestimate their sleep as compared with objective measures, although group numbers were small.

Delta values were consistent with kappa values in showing poor agreement (Table 5).

## DISCUSSION

This study found that self-reported usual sleep duration on workdays and non-workdays did not agree with actigraphically recorded actual sleep. Subjective sleep quality also showed poor agreement with the 3 individual measures of objective quality: sleep onset latency, efficiency, and wake after sleep onset. While other studies reported that an “index” of sleep quality (combining multiple domains) better reflects subjective overall sleep quality than does a single domain,^25^^,^^26^ we found that a composite objective measure did not noticeably improve agreement.

Following the lead of Mullington et al,^24^ we also examined modal sleep duration in addition to mean duration, because participants may estimate their usual sleep by using a typical night rather than by averaging sleep across nights. Unlike Mullington et al, we found that the use of modal data improved correlations with estimates of sleep duration, when compared with mean sleep. However, the overall results still failed to show strong agreement with actigraphy data.^24^

The current results therefore suggest that a 3-item questionnaire on usual sleep duration/quality does not adequately reflect objective measures of sleep as assessed using actigraphy.

Other studies that have examined agreement between subjective and objective sleep duration (using the lengthier measures of sleep quality assessment that are not realistic for epidemiologic studies) reported Pearson product-moment correlations ranging from 0.31 to 0.63.^16^^,^^32^^–^^36^ However, Pearson correlations may be inadequate for assessing validity, because a correlation measures the strength of a relationship between 2 variables but not agreement between them.^37^^,^^38^ Good correlation may occur even when agreement is poor; thus, the agreement implied in these studies may be overestimated.^37^^,^^38^ Furthermore, of the 2 studies that reported the strongest correlations, 1 was conducted in a population with advanced lung cancer and the other was conducted in a population of legally blind participants, which may limit their generalizability.^32^

To our knowledge, only 1 study has assessed agreement between subjective and objective sleep duration with statistics other than Pearson correlation. Van den Berg et al used measures of the level and direction of disagreement to compare actigraphy with estimates from sleep diaries in a large elderly population.^39^ As was the case in the present study, they found poor agreement, with one-third of participants reporting an average subjective sleep duration more than 1 hour different from their average actigraphically measured duration.^39^

Only 1 study of adults has examined agreement between sleep domains other than duration. The study of blind participants reported Pearson correlations of *r* = 0.12 for sleep onset latency and *r* = 0.06 for wake after sleep onset.^32^ Studies of young adolescent boys^40^ and young children (in which the parents completed the questionnaire)^41^ reported correlations between subjective and objective sleep onset latency of *r* = 0.49^40^ and *r* = 0.04,^41^ respectively.

There are several reasons why actual sleep may not reflect an individual’s report of their usual sleep. First, the brief questionnaire format may be unsuited to capture a multidimensional construct such as sleep and one’s subjective estimate of its parameters. In particular, the questionnaire format used in this study required respondents to provide a single value to represent their subjective estimate of usual sleep duration. However, the cognitive processes that underlie quantitative estimates of recalled sleep behavior are not clear.^42^ While questionnaire design, response formats, and social desirability can all affect responses to questionnaires, it is not known whether there are other cognitive processes that may affect how participants respond to sleep questionnaires. Participants may use mental processes such as rounding or heuristic strategies (ie, concepts of typical nights rather than average nights, adjusting for seasonal variation) when they are asked to give a single point estimate of a trait such as sleep, which has high day-to-day and seasonal variability.^1^^,^^22^^,^^23^^,^^42^ Such biases may be particularly strong when there are only 1 or 2 questions.

An additional possibility is that subjective sleep questions may not be measuring sleep habits per se but, rather, other traits that impact on the cognitive processing and heuristic shortcuts required to produce a quantitative estimate of sleep. A number of studies have reported positive associations between perceived stress and subjective, but not objective, measures of sleep quality.^23^^,^^43^^,^^44^ This study comprised self-selected participants, some of whom may have volunteered due to higher interest or concern with their sleep habits, and their self-reports may reflect this heightened concern.

Alternatively, participants may have been estimating their usual sleep after adjustment for seasonal differences. We only collected data at 1 time point, and seasonal differences in sleep have been noted.^1^

This study has several limitations. First, although actigraphy has been shown to be consistent with polysomnography among normal sleepers, it is not without limitations.^19^ Actigraphy assesses sleep by measuring motor activity, and there is the potential for actigraphy to misinterpret inactivity during wake as sleep and activity during sleep as wake.^19^^,^^45^ Anything that exaggerates, suppresses, or alters movement can result in erroneous assessment of sleep–wake.^45^

In addition, 1 week of actigraphy may not be sufficient to obtain accurate estimates of usual sleep habits. However, several studies have reported that 5 to 7 days of actigraphy was sufficient to obtain stable estimates of sleep.^22^^,^^23^

An additional limitation of this study is the relatively small numbers of participants, particularly in the less than 6 hours and greater than 8 hours sleep duration groups, despite a priori power calculations. Furthermore, the large age range of the women studied may have influenced the results, due to the association between increasing age and decreasing sleep duration and quality.^27^ The lack of information on parity, body mass index, and chronotype of participants may also be considered limitations. A replication of the study using male participants would extend the generalizability of these findings to the general population.

In summary, we found that a 3-item sleep questionnaire of the type typically used in epidemiologic studies showed poor agreement with actigraphically recorded sleep habits. These results have implications for studies examining sleep as a risk factor for morbidity and mortality. A method of accurately measuring sleep as a risk factor for long-term health outcomes remains to be determined.
